# Supplementary material for: P-TRAP: a Panicle Trait Phenotyping tool
Source: BMC Plant Biol. 2013 Aug 29;13:122. doi: 10.1186/1471-2229-13-122 (PMC3848748; doi:10.1186/1471-2229-13-122)
Supplement: Additional file 7 — P-TRAP source code. The Netbeans project that contains the source code of the application. [file 1471-2229-13-122-S7.zip › javadoc.zip!/javadoc/org-basmati-workspace/org/basmati/workspace/ImageScaler.html]

ImageScaler (WorkSpace)


JavaScript is disabled on your browser.


- Package
- Class
- Tree
- Deprecated
- Index
- Help

*org.basmati.workspace 1.0*

- Prev Class
- Next Class

- Frames
- No Frames

- All Classes

- Summary:
- Nested |
- Field |
- Constr |
- Method

- Detail:
- Field |
- Constr |
- Method


org.basmati.workspace

## Class ImageScaler

- java.lang.Object
- - org.basmati.workspace.ImageScaler

- ---

    

  ```
  public abstract class ImageScaler
  extends java.lang.Object
  ```

  The scaler of the input images in the selected project.

  Author:
  :   faroq

- - ### Constructor Summary

    Constructors

    Constructor and Description

    `ImageScaler()`
  - ### Method Summary

    Methods

    | Modifier and Type | Method and Description |
    | --- | --- |
    | `static void` | `scale(org.openide.filesystems.FileObject[] fileObject, double scalefactor)` Scale a set of images by a given scale factor. |

    - ### Methods inherited from class java.lang.Object

      `clone, equals, finalize, getClass, hashCode, notify, notifyAll, toString, wait, wait, wait`

- - ### Constructor Detail


    - #### ImageScaler

      ```
      public ImageScaler()
      ```
  - ### Method Detail


    - #### scale

      ```
      public static void scale(org.openide.filesystems.FileObject[] fileObject,
               double scalefactor)
      ```

      Scale a set of images by a given scale factor.

      Parameters:
      :   `fileObject` - {@link FileObject>
      :   `scalefactor` - double value


- Package
- Class
- Tree
- Deprecated
- Index
- Help

*org.basmati.workspace 1.0*

- Prev Class
- Next Class

- Frames
- No Frames

- All Classes

- Summary:
- Nested |
- Field |
- Constr |
- Method

- Detail:
- Field |
- Constr |
- Method
